# Commentary: Tumefactive Demyelinating Lesions as a First Clinical Event: Clinical, Imaging, and Follow-up Observations

**DOI:** 10.3389/fneur.2016.00222

**Published:** 2016-12-05

**Authors:** Tatiana Koudriavtseva, Domenico Plantone

**Affiliations:** ^1^Unit of Neurology, Multiple Sclerosis Centre, Regina Elena National Cancer Institute, IFO, Rome, Italy

**Keywords:** tumefactive demyelinating lesions, multiple sclerosis, venous vasculature, MRI, susceptibility-weighted imaging

We read with great interest the paper recently published by Jeong and colleagues exploring the long-term evolution and disease course of patients initially presenting with tumefactive demyelinating lesions (TDLs) and describing their clinical and radiographic characteristics. They found that most of TDLs evolve into multiple sclerosis (MS) or neuromyelitis optica spectrum disorders (NMOSD), albeit a minority of patients remains without a definite diagnosis, even after a careful and extensive diagnostic workup (1). The authors underlined that the current understanding of the etiology of TDLs should be revaluated upon appraisal of NMOSD because extensive brain lesions have been frequently reported as a first manifestation in NMOSD, especially in Asian populations (1).

This subject has been matter of debate in the recent years, and several papers have discussed the diagnostic challenges associated with the differential diagnosis of brain TDLs (2–6). Despite the pathogenesis of TDLs is mainly based, similar to MS, on inflammatory-demyelinating mechanisms, they present “tumor-like” radiologic characteristics, such as size greater than 2.0 cm, mass effect, and edema, atypical for classic MS lesions. Therefore, the differential diagnosis of TDLs should also include brain tumors, even in the presence of typical demyelinating lesions since MS and gliomas may, albeit rarely, coexist (7). The evolution of TDLs over time and their response to steroid therapy help to define the diagnosis but often postpone the commencement of appropriate treatments (2). Remarkably, more invasive investigations, such as brain biopsy, may sometimes be inconclusive or even lead to misdiagnosis (5).

Great efforts aimed to overcome these difficulties and to find less invasive and more reliable diagnostic tools to allow an early diagnosis of TDLs. Although some radiological characteristics on routine magnetic resonance imaging (MRI) such as an open-ring enhancement, T2-hypointense rim, peripheral restriction on diffusion-weighted imaging, and venular enhancement are considered typical for TDLs, they were not found in all cases (1–5). Even the advanced MRI techniques, such as MR spectroscopy, led to equivocal results because both normalized choline increase and *N*-acetyl-aspartate decrease were found in variable proportion in both TDLs (3, 4) and gliomas (3) with a broad overlap between these diseases. Furthermore, the relative cerebral blood volume on dynamic contrast-enhanced MRI, normalized to the respective values of the contralateral hemisphere, has been found higher in gliomas compared to TDLs (6), as well as similarly increased in both pathologies (3).

Interestingly, some radiologic findings, such as venular enhancement, edema, mass effect, and vessel-like structures running through the lesion center, support the relevance of venous involvement in TDL pathogenesis. Furthermore, one study reported signs of hemorrhage and blood stasis on and around the TDL during the venous phase on brain angiography suggesting that the presence of multiple venous dilatations can help to diagnose TDLs (4). In one well-illustrated case, there were innumerable perivenular enhancements perpendicular to the lateral ventricles within extensive bihemispheric white matter TDLs, showing a parallel temporal evolution (8). These venular enhancements are usually attributed to dilated venules draining toward distended subependymal veins (4). Enlarged blood vessels with surrounding edema and relative axonal preservation were observed also at histological examination (3, 5). Moreover, some clinical symptoms, more frequent in TDLs than in MS, such as encephalopathy, confusion, rapid memory dysfunction, seizures, stupor/coma (4, 5), and even increased intracranial pressure symptoms (1), are more compatible with venous stasis than with localized and well-defined demyelinating lesions.

Inflammatory cell infiltration and demyelination in MS have a well-known perivenular distribution involving the smaller vessels. When inflammatory processes have a greater intensity and extension, they likely determine a markedly slowed venous flow with partial thrombosis in larger venules manifesting with “tumor-like” characteristics of TDLs. Demyelinating and thrombotic diseases of the central nervous system (CNS) share common predisposing factors, such as smoking, endothelial dysfunction, platelet activation, thrombophilia, and hyper-homocysteine, all representing prothrombotic conditions (9). An increased risk of venous thromboembolism in MS reported in epidemiological studies (9) and a correlation between MS relapses and prothrombotic factors, such as antiphospholipid antibody positivity (10), support the hypothesis that the activation of coagulation system plays a role in MS pathogenesis (9). Indeed, fibrin deposition precedes and regulates the inflammatory demyelination in both experimental allergic encephalomyelitis and MS (9). Antiphospholipid positivity is even greater in NMOSD than in MS (11), and it should be noted that NMOSD usually represent a more severe disease as compared to MS and at the same time frequently associate with extensive brain lesions similar to TDLs (1). The increased antiphospholipid positivity in NMOSD indicates a raised likelihood of thrombotic phenomena in this disease, and thus, presumably in TDLs. Finally, the concept of thromboinflammation was recently applied also to ischemic stroke, defined as a thromboinflammatory disease, connecting inflammation and thrombosis through the activation of innate immunity (12).

Nevertheless, it is not surprising that venous involvement in CNS diseases has been relatively neglected since there are several practical difficulties in studying small–medium venous vasculature with routinely used MRI sequences (13). In fact, only case reports and few reviews have been published on the isolated cortical vein thrombosis so far (14), whereas there are extensive guidelines on cerebral venous thrombosis of dural sinuses and large veins (15). The application of susceptibility imaging methods, such as T2-weighted gradient-echo imaging, susceptibility-weighted imaging, and quantitative susceptibility mapping, look very promising for studying venous structure and further researches could use them in order to prove their applicability in the differential diagnosis of TDLs. Susceptibility-weighted imaging is sensitive to susceptibility differences and gives improved images of the venous system, by emphasizing deoxygenated hemoglobin (13). This allows the definition of the anatomic relationships between the venous system and the demyelinating lesions. Quantitative susceptibility mapping is a recently developed MRI technique that, by using a different approach from traditional susceptibility-weighted imaging, is able to quantify the tissue susceptibility in each pixel (13). The voxel intensity in quantitative susceptibility mapping is proportional to the underlying tissue apparent magnetic susceptibility. Interestingly, the quantitative nature of quantitative susceptibility mapping makes it an attractive tool for possible follow-up of the lesions. We believe that these new advanced MRI techniques may also improve our understanding of the pathophysiology of the TDLs, thus ameliorating the diagnostic algorithm and warranting a more specific treatment.

## Author Contributions

TK conceived and wrote the manuscript. DP wrote the manuscript.

## Conflict of Interest Statement

No conflict of interest or financial interest is reported. TK reports consulting fees from Bayer Schering and Institutional Grant from Merck Serono, Biogen Idec, Novartis, and Bayer Schering outside the submitted work. DP has nothing to declare.
